# Comparison of non-linearity correction methods for quantitative myocardial perfusion MRI

**DOI:** 10.1186/1532-429X-17-S1-P45

**Published:** 2015-02-03

**Authors:** David A Broadbent, John D Biglands, David P Ripley, Sven Plein, David L Buckley

**Affiliations:** 1 Medical Physics, University of Leeds, Leeds, UK; 2 Multidisciplinary Cardiovascular Research Centre, University of Leeds, Leeds, UK

## Background

Quantification of physiological parameters, including myocardial blood flow (MBF), by perfusion MRI relies on analysis of data that describes temporal variation of relative contrast agent concentration following bolus administration. At doses required for sufficient SNR, signal enhancement cannot be used directly as the relationship between signal intensity (SI) and the resulting concentrations in blood and myocardium is non-linear. Proposed solutions to this include separation of the AIF and myocardial data acquisition, or model based non-linearity correction.

## Methods

A simulated AIF was generated and convolved with a one-compartment (1C) model to generate myocardial data. From these data SI curves were simulated for a conventional saturation recovery (SR) sequence. SI curves were also generated with reduced dose, for a sequence with shorter saturation time (TS) and for a proton density weighted (PDw) sequence with no saturation pulse. Curves were generated assuming a range of saturation pulse efficiencies (SE). Analysis was performed using the following methods and results compared to ground truth. Signal enhancement for full dose myocardial data was analysed using the AIF from both full and reduced dose (dual-bolus), data was converted to ΔR1 using methods in the table, and PDw based conversion was also used with the AIF from the short TS sequence (dual-sequence).

**Table 1:** Model based conversion techniques.

	Data Used	Parameter(s) Estimated
Native T1	Pre-contrast T1 & SI from SR sequence	S0

Bookend T1	Pre- and post-contrast T1 & SI from SR sequence	S0 and SE

Proton Density	Pre-contrast SI from PDw and SR sequence	Baseline T1 and S0

Data used and parameters estimated in model-based conversion from signal intensity to R1.

Data from a healthy volunteer processed using the same methods was also examined.

## Results

In simulation results signal non-linearity led to substantial overestimation of MBF when using signal enhancement data from the main bolus only, or underestimation using the dual-bolus method. This was also seen in volunteer data with high (stress/rest MBF = 5.19/1.78 ml/min/ml) and low (1.72/0.33) results for these analyses.

In the simulation study PDw or bookend T_1_ based conversion eliminated these errors for realistic SE values, and yielded comparable results in vivo (2.98/1.00 and 2.72/0.93 respectively). Native T_1_ based conversion exhibited strong SE dependence due to variation of baseline SI with SE, with conversion failure occuring above a threshold over-saturation value. Conversion failure was also observed in volunteer data, consistent with the 1.3% over-saturation estimated during bookend T_1_ analysis.

In the simulation data the dual-sequence technique was robust for perfect or under-saturation, but over-saturation of more than around 1% led to small systematic errors. However, differences between dual-sequence results for the volunteer (1.64/0.69) and PDw or bookend T_1_ based conversion results were substantially larger than the simulations predicted, and so further investigation is required to understand this discrepancy.

## Conclusions

Application of methods to account for signal non-linearity in myocardial perfusion MRI can be strongly influenced by small imperfections in saturation efficiency and so the robustness of the chosen method should be considered in any study of absolute quantification of perfusion data.

## Funding

David Broadbent is funded by National Institute for Health Research Doctoral Research Fellowship NIHR-DRF-2012-05-155.

**Figure 1:**
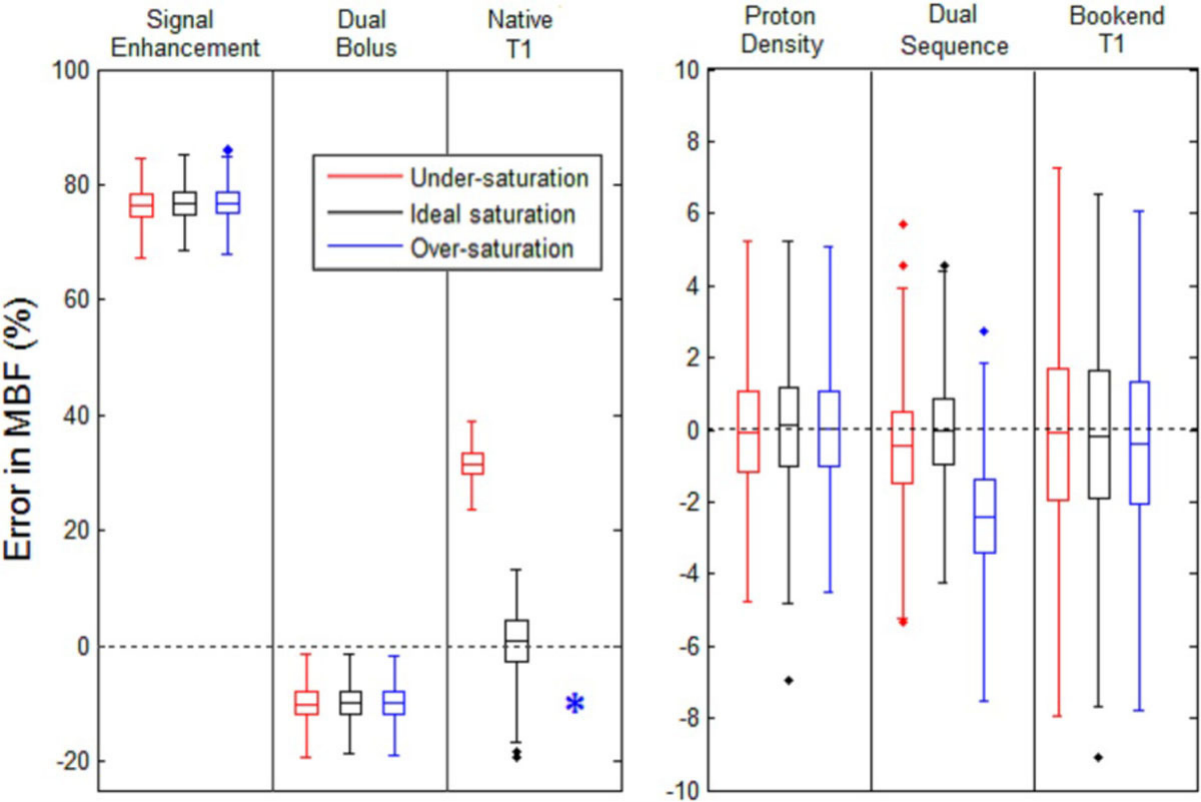
Errors in MBF estimation from simulation results for perfect saturation (SE = 1) and 2.5% over- and under-saturation (SE = 1.025 and 0.975 respectively). Results are separated into two figures due to the substantially larger range of errors for signal enhancement, dual bolus and native T1 based correction in comparison to the other results. * conversion failed for native T1 based correction as at the peak of the AIF no positive value of R1 satisfied the model determined by the baseline T1 and SI data. Ground truth MBF was 0.83 ml/min/ml and the distribution (extracellular) volume was 25%.

